# An LC‐MS/MS Method for Quantitative Analysis of Regorafenib and Exploration of Its Intracellular Exposure in Colorectal Cancer Cells

**DOI:** 10.1155/ianc/5522314

**Published:** 2026-10-29

**Authors:** Xinran Wang, Wenwen Cai, Zhijun Liu, Jianguo Sun, Zhenhui Jiang, Liya Ye, Zhengyan Liang, Zhipeng Wang, Shouhong Gao, Wansheng Chen

**Affiliations:** ^1^ Department of Pharmacy Second Affiliated Hospital of Naval Medical University (Shanghai Changzheng Hospital) Shanghai 200003 China; ^2^ Department of Hematology Myeloma & Lymphoma Center Second Affiliated Hospital of Naval Medical University (Shanghai Changzheng Hospital) Shanghai 201203 China; ^3^ College of Traditional Chinese Medicine Shanghai University of Traditional Chinese Medicine Shanghai 201203 China shutcm.edu.cn

**Keywords:** colorectal cancer, intracellular exposure, mitochondrial fraction, nuclear fraction, regorafenib, UHPLC-MS/MS

## Abstract

**Objective:**

Regorafenib (Reg) is a multikinase inhibitor for treating metastatic colorectal cancer (CRC); however, plasma concentrations may not fully predict clinical response. This study aimed to develop and validate an LC‐MS/MS method for quantifying Reg in CRC cells and to preliminarily characterize its temporal and spatial distribution.

**Methods:**

Separation of Reg and the internal standard (selinexor) was achieved using an Agilent Poroshell 120 EC‐C18 column (3.0 × 100 mm, 2.7 μm) with isocratic elution using acetonitrile and 0.1% aqueous formic acid (70:30, v/v). Detection was performed in multiple reaction monitoring mode using an Agilent 1260‐6460 UHPLC‐MS/MS system. HCT116 and SW480 cells were treated with Reg at their respective IC50 concentrations for 12, 24, 36, and 48 h, and Reg exposure in whole‐cell, nuclear, and mitochondrial fractions was measured.

**Results:**

The UHPLC‐MS/MS method showed good linearity (*R* ^2^ > 0.99) and a lower limit of quantification of 1.0 ng/mL. Precision (intraday RSD% < 11.39%; interday RSD% < 9.11%) and accuracy (RE% within ±6.80%) were within the acceptance criteria. The recovery ranged from 86.03% to 106.41%, and consistent matrix effects were observed. Reg exposure in whole‐cell samples increased in a time‐dependent manner, while nuclear and mitochondrial fraction‐associated exposures showed distinct temporal patterns between the two CRC cell lines.

**Conclusion:**

The validated UHPLC‐MS/MS method enables sensitive and reliable quantification of Reg in CRC cells and isolated subcellular fractions. The observed temporal differences in intracellular and fraction‐associated Reg exposure may be associated with cellular response and may support future exposure–response assessments beyond plasma‐based pharmacokinetics.

## 1. Introduction

According to a cancer report released by the National Cancer Center of China in January 2024, approximately 517,000 new cases of colorectal cancer (CRC) were diagnosed in 2022, accounting for 20.3% of all new cancer cases. The number of CRC‐related deaths was approximately 86,200, representing 24.0% of all cancer‐related mortalities [1]. Globally, CRC ranks as the second most commonly diagnosed malignancy and the third leading cause of cancer‐related death, highlighting the urgent need for effective treatment strategies [2]. Data from the U.S. Surveillance, Epidemiology, and End Results (SEER) database (2014–2020) indicate that advanced‐stage CRC accounts for 23% of all CRC cases, whereas the 5‐yearoverall survival rate of these patients remains low at only 15.7% [3]. Regorafenib (Reg, BAY73‐4506, Stivarga) is an oral multikinase inhibitor belonging to the class of phenylurea derivatives [4]. It targets angiogenesis‐related receptors (VEGFR1‐3, TIE2), stromal‐related receptors (PDGFR‐β, FGFR), and oncogenic receptor tyrosine kinases (KIT, RET, RAF) [5]. Clinically, Reg is indicated for the treatment of metastatic CRC (mCRC), gastrointestinal stromal tumors, and hepatocellular carcinoma [6]. As the first multikinase inhibitor demonstrated survival benefits in mCRC following failure of standard therapies, Reg was approved by the Food and Drug Administration (FDA) for this indication in 2012 [7]. By inhibiting multiple kinase targets, Reg delays tumor progression and improves patient survival, establishing it as an important third‐line or later treatment option for patients with resistant disease [8]. In an international phase III CORRECT trial, Reg significantly extended overall survival in patients with advanced CRC who had progressed after standard therapy [9]. However, considerable interpatient variability in treatment response has been reported among Reg‐treated mCRC patients, underscoring the need for more reliable predictors of efficacy. Traditional pharmacokinetic analysis mainly relies on plasma concentrations, which may not accurately reflect drug exposure at the site of action. Previous clinical pharmacokinetic studies did not demonstrate a clear exposure–efficacy relationship for Reg alone in CRC treatment, although the summed concentrations of Reg and its two major metabolites showed a preliminary positive association with progression‐free survival [10]. These findings suggest that plasma exposure alone may be insufficient for individualized dose optimization. Recent studies have proposed that intracellular drug concentrations, including drug exposure in subcellular fractions,for example, the nucleus and mitochondria, may provide complementary information for evaluating drug response [11, 12]. These observations prompted us to explore the intracellular and fraction‐associated exposure of Reg in CRC cells.

In this study, an ultra‐high performance liquid chromatography–tandem mass spectrometry (UHPLC‐MS/MS) method was developed and validated to quantify Reg in HCT116 and SW480 CRC cells and isolated subcellular fractions. The temporal distribution of Reg in whole‐cell, nuclear, and mitochondrial fractions was investigated at 12, 24, 36, and 48 h. By integrating intracellular exposure data with cell viability results, this study aimed to provide preliminary evidence for the potential relationship between intracellular Reg exposure and cellular response and to support future exposure–response investigations beyond plasma‐based pharmacokinetics.

## 2. Materials and Methods

### 2.1. Chemicals and Reagents

The Reg standard (lot: MB5106, purity > 99%) was purchased from Dalian Meilun Biotechnology Co., Ltd. (Shanghai, China). Selinexor (internal standard, IS; lot: C13J11L117641, purity > 99%) was obtained from Yuanye Bio‐Technology Co., Ltd. (Shanghai, China). HPLC‐grade methanol and acetonitrile were obtained from Merck (Darmstadt, Germany). HPLC‐grade formic acid was purchased from McLean Biochemical Technology Co., Ltd. (Shanghai, China). Ultrapure water was used throughout the study. Cell fractionation kits for the isolation of cell nucleus and mitochondria were procured from Abbkine Scientific Co., Ltd. (Wuhan, China).

### 2.2. Cell Culture

The human colorectal cancer cell line HCT116 was a stored cell line maintained in our laboratory. The human colon cancer cell line SW480 was purchased from Xinyu Biological Technology Co., Ltd. (Shanghai, China). HCT116 cells were maintained in McCoy’s 5A medium, and SW480 cells were cultured in Leibovitz’s L‐15 medium; both media were supplemented with 10% heat‐inactivated fetal bovine serum and 1% penicillin–streptomycin. HCT116 cells were incubated at 37°C in a 5% CO_2_ atmosphere, and SW480 cells were cultured at 37°C without CO_2_. The culture medium was replaced every 2–3 days. Upon reaching 80%–85% confluence, the cells were either passaged or harvested for subsequent experiments.

### 2.3. LC‐MS/MS Instrumentation

Chromatographic analysis was performed using an Agilent 1290 series UHPLC system, equipped with an online degasser, a binary pump, an autosampler, and a thermostated column compartment. The UHPLC system was coupled to an Agilent 6460A triple quadrupole mass spectrometer with an electrospray ionization (ESI) source (Agilent Technologies, Santa Clara, USA). Data acquisition and processing were conducted using Agilent MassHunter Workstation Software (Version 6.00).

### 2.4. Liquid Chromatographic Conditions

Separation was achieved using an Agilent Poroshell 120 EC‐C_18_ column (3.0 × 100 mm, 2.7 μm) maintained at 35°C. The mobile phase consisted of 0.1% formic acid in water (A) and acetonitrile (B). Isocratic elution was performed with a mixture of 30% A and 70% B. The total run time was 3.65 min, with a flow rate of 0.45 mL/min.

### 2.5. Mass Spectrometry Conditions

ESI was conducted in positive ionization mode with the capillary voltage set to +4500 V. Data acquisition was performed in multiple reaction monitoring (MRM) mode. Nitrogen was employed as the desolvation, sheath, and nebulizing gas. The nebulizer pressure was set to 45 psi, and the desolvation gas was heated to 320°C with a flow rate of 10 L/min. The sheath gas temperature was set to 300°C with a flow rate of 12 L/min. High‐purity nitrogen was used as the collision gas at a pressure of 0.2 MPa. The product ion and fragment structures of Reg and IS are shown in Figure 1. The optimized mass spectrometry parameters for Reg and the IS are summarized in Table 1.

**FIGURE 1:**
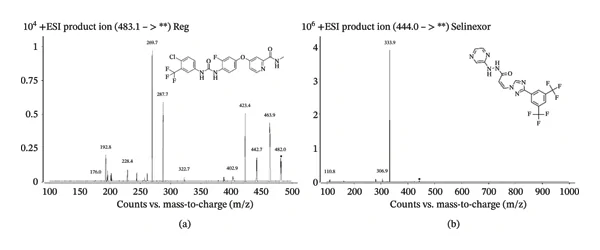
Product ion chromatograms and fragment structures of Reg (a) and IS (b).

**TABLE 1:** MS parameters of Reg and IS.

Analyte	Ionization mode	Molecular weight	Precursor (m/*z*)	Product ion (m/*z*)	Fragmentor (V)	Ce (V)
Reg	+	482.81	483.1	270.1	180	28
Selinexor (IS)	+	443.31	444.0	334.0	130	15

### 2.6. Preparation of Standard and Quality Control (QC) Solutions

Stock solutions of Reg (1000.0, 100.0, and 10.0 μg/mL) were prepared by dissolving 2.0 mg of the standard in methanol. Aliquots of these stock solutions were transferred to 1.5‐mL tubes and stored at −80°C. Working solutions were subsequently prepared by diluting with methanol and were further spiked into blank cell matrix (v:v,1:9) to generate calibration standards at concentrations of 1.0, 2.0, 10.0, 50.0, 200.0, 500.0, 750.0, and 1000.0 ng/mL. QC samples at low, medium, and high concentrations (2.0, 500.0, and 750.0 ng/mL, respectively) were prepared independently in the same manner. The IS stock solution (1.0 mg/mL) was stored at −80°C and diluted with methanol to a concentration of 200.0 ng/mL immediately before use.

### 2.7. Cell Viability Assay

HCT116 and SW480 cells were seeded into 96‐well plates at a density of 5 × 10^3^ cells per well and cultured overnight in McCoy’s 5A and Leibovitz’s L‐15 medium, respectively. The cells were treated with Reg at concentrations ranging from 0 to 2 μM (0, 0.005, 0.02, 0.05, 0.1, 0.2, 0.5, and 1, 2) in their respective medium for 48 h. Cell viability was assessed using a Cell Counting Kit‐8 (CCK‐8) according to the manufacturer’s instructions. Absorbance was measured at 450 nm using a SYNERGY microplate reader (BioTek, Winooski, USA). Cell viability was calculated as follows: (OD treated group − OD blank group)/(OD control group − OD blank group) × 100%.

### 2.8. Cell Sample Collection and Pretreatment

HCT116 and SW480 cells were seeded in 6‐well plates at 1 × 10^6 cells per well and cultured for 24 h. Upon reaching 80%–90% confluence, the medium was replaced with fresh McCoy’s 5A or L‐15 medium containing Reg at the IC_50_ values, respectively, and the cells were incubated for 12, 24, 36, or 48 h. After incubation, cells were harvested, counted, and washed twice with ice‐cold phosphate‐buffered saline (PBS). Nuclear and mitochondrial fractions were isolated using commercial fractionation kits (ExKineTM Nuclear and Mitochondrial Isolation Kits) according to the manufacturer’s instructions. All fractionation procedures were performed using precooled buffers and completed as rapidly as possible under cold conditions to minimize postharvest redistribution of Reg. For whole‐cell samples, cells were extracted with 500 μL of methanol on ice for 2 min, followed by evaporation of the supernatant. The dried residue was resuspended in 200 μL of methanol and centrifuged at 14,500 × g for 10 min. The resulting supernatant was mixed with 300 μL of methanol containing 100 ng/mL IS, vortexed for 2 min, and then centrifuged at 14,500 × g for 10 min. For nuclear and mitochondrial fractions, 100 μL of each extract was mixed with 300 μL of methanol containing the IS (100 ng/mL), vortexed for 2 min, and then centrifuged at 14,500 × g for 10 min. The supernatant was transferred to a sample vial for LC‐MS/MS analysis. The measured Reg levels in nuclear and mitochondrial samples should be interpreted as Reg recovered from isolated fractions under the present operational procedure rather than as direct evidence of absolute in situ subcellular localization. All experiments were performed in triplicate.

### 2.9. Method Validation

The developed method was validated in accordance with the bioanalytical guidelines provided by the U.S. FDA and the Chinese Pharmacopoeia (2020 edition). The following parameters were validated: specificity, linearity, precision, accuracy, matrix effect, recovery, carry‐over, dilution integrity, and stability [13, 14].

Specificity was evaluated by analyzing six different lots of blank matrix, blank matrix spiked with the IS, lower limit of quantification(LLOQ) samples, and processed real samples. The method was deemed acceptable if the response of any interfering peak was ≤ 20% of the LLOQ response and ≤ 5% of the IS response.

Linearity was assessed by analyzing three independently prepared calibration curves daily over a minimum of 2 days. Calibration curves were constructed using a weighted linear least‐squares regression model by plotting the peak area ratio of analyte to IS against the nominal analyte concentration. TheLLOQ was defined as the lowest concentration on the calibration curve. The back‐calculated concentration of each calibration standard was required to be within ±15% of the nominal value (±20% for the LLOQ).

Intraday precision and accuracy were determined by analyzing five replicates of QC samples at three concentration levels (low, medium, high) in a single run. Interday precision and accuracy were assessed by analyzing the same QC levels over a minimum of three separate runs. For both intraday and interday measurements, precision (RSD%) was required to be ≤ 15%, and accuracy(RE%) was required to be within ±15% compared to the nominal value.

Extraction recovery was determined by comparing the peak areas of analytes spiked into the matrix before extraction with those spiked into the blank matrix extract postextraction at the same concentration. The matrix effect was evaluated by comparing the peak areas of post‐extraction spiked samples with those of solvent‐substituted standard solutions at equivalent concentrations. RSD% of the matrix effect and extraction recovery factors ≤ 15% were considered acceptable, indicating consistent ion suppression or enhancement from the matrix.

Carry‐over was assessed by injecting a blank sample immediately following the injection of an upper limit of quantification (ULOQ) standard. The response of the analyte in the blank sample was required to be ≤ 20% of the LLOQ and ≤ 5% of the IS.

Dilution integrity was evaluated by diluting samples with concentrations above the ULOQ with blank matrix into the calibration range. Five replicates per dilution factor were analyzed, and the precision was required to be ≤ 15% and accuracy to be within ±15%.

Stability was investigated using QC samples at low, medium, and high concentrations under the following conditions: bench‐top stability at room temperature for 4 h, processed sample stability in the autosampler (0, 12, and 24 h), three freeze–thaw cycles, and long‐term storage at −80°C for 1 month. Stability was confirmed if the measured concentration deviated not more than ±15% from the nominal concentration.

### 2.10. Data Analysis

All data were analyzed using GraphPad Prism software (Version 9.0, GraphPad Software, San Diego, CA, USA). Intergroup comparisons were performed using an unpaired Student’s *t*‐test or a one‐way analysis of variance (ANOVA). A *p* value of < 0.05 was considered statistically significant.

To explore the potential relationship between intracellular Reg exposure and cellular response, the cell viability data and intracellular exposure data were integrated descriptively. Because the cell viability assay was performed as a 48‐h concentration–response experiment, whereas intracellular and fraction‐associated Reg exposure was measured over time at the corresponding IC50 concentrations, the two datasets were not fully matched for formal exposure–response modeling. Therefore, the integrated analysis was considered exploratory.

## 3. Results and Discussion

### 3.1. The Optimization of UHPLC‐MS/MS Conditions

To improve the selectivity and sensitivity of the method, the ionization of the analyte in atmospheric pressure chemical ionization (APCI) and ESI sources was evaluated by comparing the response of the analyte under positive and negative ionization modes. It was found that ionization of Reg and the IS was higher in the positive ionization mode in the ESI source.

When selecting the chromatographic column, an appropriate one was required to ensure proper retention and separation of the analyte. Consequently, this study evaluated various columns, including Agilent Poroshell 120 EC‐C18 (3.0 × 100 mm, 2.7 μm), Agilent ZORBAX SB‐C8 (2.1 × 150 mm, 5 μm), and Waters XBridge C18 (2.1 × 100 mm, 3.5 μm). Ultimately, the Agilent Poroshell 120 EC‐C18 (3.0 × 100 mm, 2.7 μm) was chosen as the most suitable column, providing better peak shape, retention time, and separation of Reg and the IS. The effects of column temperature (25°C, 30°C, 35°C, and 40°C) on the retention time of Reg were investigated. Results indicated that 35°C was optimal for Reg retention, resulting in a retention time of 3 min.

Additionally, mobile phases with various additives, including methanol, acetonitrile, and aqueous solutions containing ammonium acetate, formic acid, and ammonium formate, were evaluated. The results indicated that acetonitrile generated stronger signals, whereas aqueous solutions containing 0.1% formic acid significantly enhanced ionization efficiency. This improvement in ionization led to better peak shape and response for Reg and minimized matrix effects.

### 3.2. Sample Pretreatment

Sample pretreatment is a critical step for removing matrix interferents, thereby improving recovery and stabilizing the matrix effects. Several methods for removing proteins and other matrix interferents were evaluated, including protein precipitation, solid‐phase extraction, and liquid‐liquid extraction. The results showed that both solid‐phase extraction and liquid‐liquid extraction resulted in low recoveries for Reg (both < 40%). In contrast, protein precipitation using methanol provided higher recovery (> 85%) and more stable matrix effects. After comparing the influence of different methanol‐to‐sample ratios on recovery and matrix effects, a sample‐to‐methanol ratio of 1:3 (V:V) was selected, which achieved optimal recovery and minimized matrix effects.

### 3.3. Method Validation

#### 3.3.1. Specificity

The retention times of Reg and the IS were 2.2 min and 1.5 min, respectively. A comparison of analyte responses in blank, LLOQ, and real samples showed no interference at the retention times of Reg and the IS (Figure 2).

**FIGURE 2:**
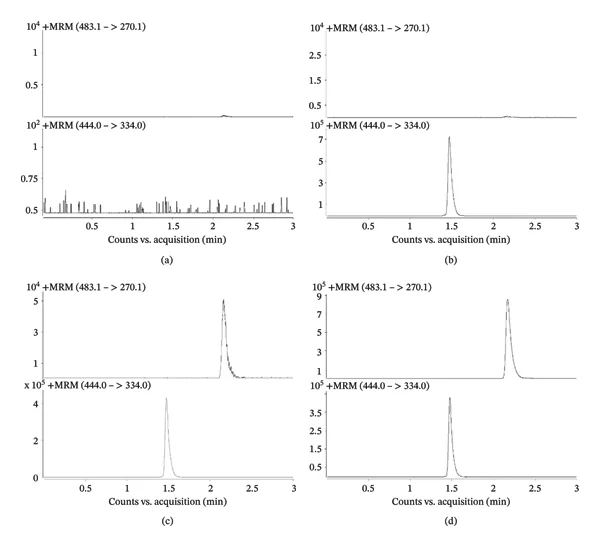
Representative MRM chromatograms showing the specificity of Reg (a) blank sample; (b) blank sample spiked with IS; (c) LLOQ sample; (d) real sample.

#### 3.3.2. Linearity of Calibration Curves and LLOQ

The working solutions were added to blank cell matrix and diluted into a series of calibration standards for Reg (1.0, 2.0, 10.0, 50.0, 200.0, 500.0, 750.0, and 1000.0 ng/mL). Calibration curves were constructed by plotting the peak area ratio of the analyte to the IS against the nominal analyte concentrations and were fitted using a weighted least‐squares model (weighting factor 1/x^2). As shown in Table 2, the calibration curve showed good linearity, with an *R* ^2^ value greater than 0.99. The LLOQ of Reg was established at 1.0 ng/mL. After pretreatment, six parallel measurements of LLOQ samples yielded an RSD% below 4.24%, and the back‐calculated concentrations met the validation criteria (Table 3).

**TABLE 2:** Linear regression parameters of Reg.

Analyte	Regression type	Linear range (ng/mL)	Regression equation	*R* ^2^
Reg	Linearity	2.0–1000.0	*y* = 0.003044χ + 0.007005	0.9974

**TABLE 3:** Assessment of LLOQ of Reg (*n* = 6).

Analyte	Nominal concentration (ng/mL)	Measured concentration (ng/mL, mean ± SD)	RSD (%)	RE (%)
Reg	1.0	1.13 ± 0.05	4.24	12.52

#### 3.3.3. Recovery and Matrix Effect

The extraction recovery of Reg ranged from 86.03% to 106.41%, with RSD% values below 3.00%, indicating that the methanol‐based protein precipitation procedure provided reproducible extraction efficiency. The matrix effects of Reg ranged from 84.89% to 88.21%, indicating moderate ion suppression in the cell matrix. However, the matrix effect was consistent across low, medium, and high QC levels, with RSD% values below 5.69%. Because matrix‐matched calibration standards and QC samples were used and the analyte response was normalized with the IS, this reproducible matrix effect was considered acceptable for quantitative analysis. In addition, the intraday and interday precision and accuracy met the acceptance criteria, further supporting the reliability of the method despite the observed ion suppression (Tables 4 and 5). Standard addition or dilution‐based evaluation may further characterize matrix effects in future applications involving more complex clinical or patient‐derived samples.

**TABLE 4:** Extraction recovery and matrix effect of Reg (*n* = 3).

Component	Nominal concentration (ng/mL)	Recovery		Matrix effect	
		Mean% ± SD	RSD (%)	Mean% ± SD	RSD (%)
Reg	2.0	105.71 ± 2.11	2.00	84.89 ± 4.83	5.69
	500.0	106.41 ± 3.18	3.00	87.44 ± 2.35	2.73
	750.0	86.03 ± 0.54	0.63	88.21 ± 0.40	0.44

**TABLE 5:** The intraday and interday precision and accuracy of Reg (*n* = 5).

Analyte	Nominal concentration (ng/mL)	Intraday			Interday		
		Measured concentration (Mean ± SD)	Precision (RSD %)	Accuracy (RE %)	Measured concentration (Mean ± SD)	Precision (RSD %)	Accuracy (RE %)
Reg	2.0	1.92 ± 0.21	11.39	3.5	1.99 ± 0.18	9.11	6.80
	500.0	521.25 ± 24.41	4.68	4.25	516.48 ± 26.02	5.04	3.30
	750.0	758.71 ± 32.98	4.35	1.16	741.06 ± 25.47	3.45	1.19

#### 3.3.4. Intraday and Interday Precision and Accuracy

Following the guidelines, three lots of spiked samples at low, medium, and high concentrations were prepared and tested in five replicates. The intraday precision had an RSD% range of 4.35%–11.39%, and the intraday accuracy showed an RE% range of 1.16%–4.25%. Interday precision and accuracy showed RSD% of 3.45%–9.11% and RE% of 1.19%–6.80%, respectively, meeting the criteria (Table 5).

#### 3.3.5. Stability

The stability of Reg was evaluated across varying concentrations: low, medium, and high. The analyte remained stable at room temperature for up to 4 h and in the autosampler for 24 h. Long‐term storage and freeze–thaw stability also demonstrated satisfactory results, with deviations (RE%) for analyte within ±15%. These findings confirm that the stability of Reg under various conditions meets the requirements (Tables 6 and 7).

**TABLE 6:** Results of autosampler stability of Reg (*n* = 3).

Component	Nominal concentration (ng/mL)	0 h			12 h			24 h		
		Measured concentration (ng/mL, mean ± SD)	Precision (RSD%)	Accuracy (RE%)	Measured concentration (ng/mL, mean ± SD)	Precision (RSD%)	Accuracy (RE%)	Measured concentration (ng/mL, mean ± SD)	Precision (RSD%)	Accuracy (RE%)
Reg	2.0	1.97 ± 0.12	6.33	1.47	1.98 ± 0.18	9.31	0.82	1.95 ± 0.12	5.93	2.27
	500.0	526.13 ± 14.93	2.83	−5.22	507.64 ± 9.73	1.92	−1.53	537.29 ± 30.37	5.65	7.46
	750.0	738.37 ± 8.69	1.18	1.54	768.60 ± 18.65	2.43	−2.48	741.55 ± 22.82	3.08	1.13

**TABLE 7:** Results of room temperature stability, three freeze–thaw cycles stability, and long‐term stability of Reg (*n* = 3).

Component	Nominal concentration (ng/mL)	Room temperature, 4 h			Three freeze–thaw cycles			Long‐term stability		
		Measured concentration (ng/mL, mean ± SD)	Precision (RSD%)	Accuracy (RE%)	Measured concentration (ng/mL, mean ± SD)	Precision (RSD%)	Accuracy (RE%)	Measured concentration (ng/mL, mean ± SD)	Precision (RSD%)	Accuracy (RE%)
Reg	2.0	1.84 ± 0.11	5.90	8.17	1.91 ± 0.06	2.97	4.59	1.90 ± 0.20	1.07	4.81
	500.0	503.42 ± 10.16	2.02	−0.68	536.42 ± 28.98	5.40	−7.28	495.21 ± 14.08	2.84	0.96
	750.0	756.71 ± 33.89	4.78	−0.89	738.69 ± 6.60	0.89	1.51	763.55 ± 7.29	0.96	−1.81

### 3.4. Spatial Intracellular Exposure of Reg and Exploratory Association With Cellular Response

To investigate the intracellular exposure characteristics of Reg in CRC cells, HCT116 and SW480 cells were first treated with different concentrations of Reg for 48 h to evaluate cell viability. As shown in Figure 3, Reg inhibited the viability of both HCT116 and SW480 cells in a concentration‐dependent manner. The IC_50_ values were 0.11 μM for HCT116 cells and 0.57 μM for SW480 cells, indicating that HCT116 cells were more sensitive to Reg treatment than SW480 cells.

**FIGURE 3:**
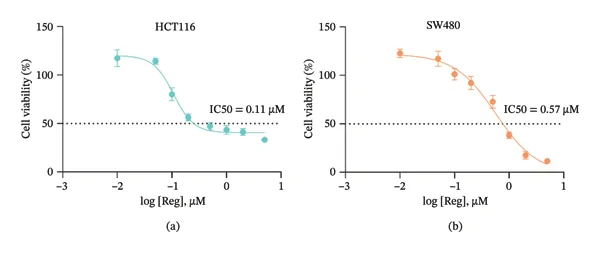
Cell viability of HCT116 cells and SW480 cells treated with different concentrations of Reg.

Based on the IC_50_ values, HCT116 and SW480 cells were subsequently treated with Reg at 0.11 μM and 0.57 μM, respectively, for 12, 24, 36, and 48 h. Reg concentrations in whole‐cell, nuclear, and mitochondrial fractions were quantified using the validated UHPLC‐MS/MS method. As shown in Figure 4, Reg exposure in whole‐cell samples increased over time in both cell lines, suggesting progressive intracellular accumulation during treatment. Reg exposure recovered from nuclear fractions also showed a generally time‐dependent increase, although the accumulation patterns differed between HCT116 and SW480 cells. In HCT116 cells, nuclear fraction‐associated Reg exposure level continued to increase over time, whereas in SW480 cells, nuclear fraction‐associated exposure level increased initially but decreased after 36 h. In mitochondrial fractions, Reg exposure level displayed a nonlinear temporal pattern rather than a continuous increase, suggesting that mitochondrial fraction‐associated Reg exposure may vary dynamically during treatment.

**FIGURE 4:**
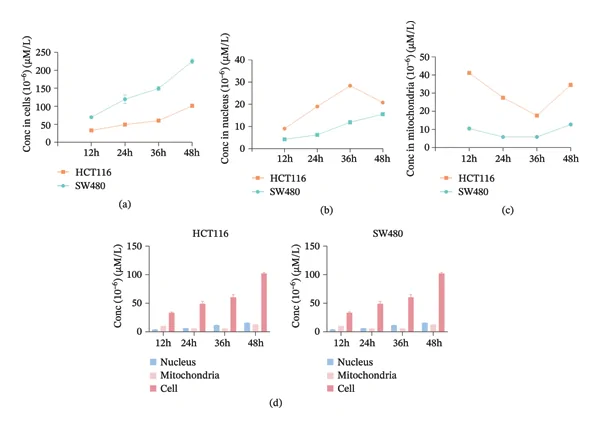
Temporal and spatial variations of Reg exposure levels in whole‐cell, nuclear, and mitochondrial fractions at 12, 24, 36, and 48 h. A parallel experiment was performed to measure cell number, and Reg concentrations were normalized accordingly (*n* = 3). (a) Concentration–time curve of Reg in whole‐cell samples of HCT116 and SW480 cells. (b) Concentration–time curve of Reg in nuclear fractions of HCT116 and SW480 cells. (c) Concentration–time curve of Reg in mitochondrial fractions of HCT116 and SW480 cells. (d) Comparison of Reg concentrations in whole‐cell, nuclear, and mitochondrial fractions of HCT116 and SW480 cells.

It should be noted that the nuclear and mitochondrial Reg concentrations measured in this study represent Reg recovered from isolated fractions. Although commercial fractionation kits were used and samples were processed rapidly under cold conditions, protein marker validation of fraction purity was not performed. In addition, potential redistribution of Reg during the fractionation procedure cannot be completely excluded. Therefore, these data should be interpreted as fraction‐associated Reg exposure rather than definitive evidence of absolute subcellular localization. Further studies using compartment‐specific protein markers and optimized rapid quenching procedures are needed to validate the purity of isolated fractions and to confirm the subcellular distribution of Reg.

To further explore the relationship between intracellular Reg exposure and cellular response, the cell viability data and intracellular exposure profiles were integrated descriptively. The lower IC_50_ value observed in HCT116 cells, together with the sustained increase in whole‐cell and nuclear fraction‐associated Reg exposure, suggests a potential association between intracellular Reg accumulation and cellular sensitivity to Reg. However, the cell viability assay was performed as a 48‐h concentration–response experiment, whereas the intracellular distribution experiment was conducted at the corresponding IC_50_ concentrations across multiple time points. Therefore, these datasets were not fully matched for formal exposure–response modeling. The current analysis should be considered exploratory and cannot establish a direct causal relationship between fraction‐associated Reg concentration and anticancer efficacy.

Among the analyzed fractions, whole‐cell and nuclear fraction‐associated Reg exposure showed relatively consistent time‐dependent increases, whereas mitochondrial fraction‐associated exposure showed a more variable pattern. These findings suggest that intracellular and fraction‐associated Reg exposure may contribute to the cellular response to Reg. Nevertheless, based on the current data, it is not possible to determine which specific subcellular fraction is the dominant determinant of Reg efficacy. Further studies using matched concentration–time exposure data, time‐course viability assays, validated fractionation, and organelle‐specific functional assays are required to clarify the compartment most closely associated with Reg activity.

The different nuclear fraction‐associated accumulation patterns between HCT116 and SW480 cells may reflect cell line‐specific differences in drug uptake, efflux capacity, or nuclear‐associated drug distribution. In addition, previous studies have reported that Reg can interact with membrane structures, and such membrane‐associated interactions may partly contribute to its fraction‐associated distribution pattern [15]. However, nuclear transporters and nuclear membrane‐associated mechanisms were not directly examined in this study. Therefore, this explanation remains hypothetical and requires further validation.

Similarly, the nonlinear changes in mitochondrial fraction‐associated Reg exposure may be related to dynamic alterations in mitochondrial‐associated drug distribution. Previous studies have suggested that Reg may affect mitochondrial function and cellular energy metabolism, including mitochondrial impairment and AMPK activation [16]. However, ATP production, mitochondrial membrane potential, AMPK activation, and OXPHOS activity were not measured in the present study. Thus, the possible involvement of mitochondrial dysfunction or energy stress should be interpreted as a hypothesis rather than a confirmed mechanism.

From a translational perspective, intracellular quantification of Reg may complement conventional plasma pharmacokinetic analysis by providing information on drug exposure closer to the site of action. Plasma concentrations mainly reflect systemic exposure but may not fully represent drug accumulation within tumor cells or isolated subcellular fractions. The UHPLC‐MS/MS method established in this study may provide a technical basis for future studies using patient‐derived CRC cells, tumor organoids, or clinically accessible surrogate cellular models to evaluate intracellular Reg exposure. Such approaches may help to explore interindividual variability in drug response, improve exposure–response assessment, and support individualized treatment strategies. Nevertheless, the present study remains preclinical, and further validation using clinical samples, matched clinical response data, and standardized sample processing procedures is required before intracellular Reg measurement can be applied to therapeutic drug monitoring or individualized treatment strategies.

Several limitations of this study should be acknowledged. First, the cell viability assay and intracellular exposure experiment were not performed using a fully matched concentration–time design; therefore, the relationship between intracellular Reg exposure and cell viability could only be evaluated exploratorily, and definitive exposure–response modeling could not be established. Second, although Reg concentrations were measured in isolated nuclear and mitochondrial fractions, protein marker validation of fraction purity was not performed, and potential redistribution of Reg during fractionation cannot be completely excluded. Third, although different temporal patterns of Reg exposure were observed in nuclear and mitochondrial fractions, this study did not include functional assays of nuclear transport, mitochondrial activity, ATP production, AMPK activation, or OXPHOS. Fourth, Reg concentrations in the culture medium were not measured; therefore, cellular uptake fraction and extracellular–intracellular mass balance could not be determined. Future studies should include protein marker validation, culture medium quantification, matched time‐course viability assays, standard addition or dilution‐based matrix‐effect evaluation where appropriate, and organelle‐specific functional validation to clarify the relationship between Reg uptake, intracellular distribution, and cellular response.

## 4. Conclusion

In this study, a simple, rapid, and sensitive UHPLC‐MS/MS method was developed and validated for the quantification of Reg in CRC cells and isolated subcellular fractions. The method showed satisfactory linearity, precision, accuracy, recovery, matrix effect, and stability. Reg inhibited the growth of HCT116 and SW480 cells in a concentration‐dependent manner. In addition, Reg concentration showed temporal differences in whole‐cell, nuclear, and mitochondrial fractions, suggesting that intracellular and fraction‐associated exposure may be associated with cellular response. However, due to the exploratory design of the current analysis and the lack of fraction purity validation, further studies using validated subcellular fractionation, extracellular medium quantification, matched exposure–response designs, and functional assays are needed to clarify the relationship between Reg intracellular distribution and its anticancer activity.

## Author Contributions

Xinran Wang: data curation, investigation, methodology, and writing–original draft. Wenwen Cai: methodology and writing–original draft. Zhijun Liu: assistance with original draft writing and editing, conceptualization, and resources. Jianguo Sun: technical support and conceptualization. Zhenhui Jiang, Liya Ye, and Zhengyan Liang: methodology and validation. Zhipeng Wang: formal analysis, writing–review and editing, supervision, conceptualization, and funding acquisition. Shouhong Gao: project administration, conceptualization, and funding acquisition. Wansheng Chen: supervision, project administration, resources, conceptualization, and funding acquisition.

## Funding

This research received funding from the National Natural Science Foundation of China (U23A20512, 82530119, 82204819) and the “Shenlan” Talent Project of Naval Medical University (SL63).

## Conflicts of Interest

The authors declare no conflicts of interest.

## Data Availability

All of the data generated or analyzed during this study are available from the corresponding author on reasonable request.
